# UV-Blocking, Transparent, and Antioxidant Polycyanoacrylate Films

**DOI:** 10.3390/polym12092011

**Published:** 2020-09-03

**Authors:** Ana Isabel Quilez-Molina, Lara Marini, Athanassia Athanassiou, Ilker S. Bayer

**Affiliations:** 1 Smart Materials, Istituto Italiano di Tecnologia, 16163 Genova, Italy; lara.marini@iit.it (L.M.); Athanassia.Athanassiou@iit.it (A.A.); 2 Dipartimento di Informatica, Bioingenieria, Robotica e Ingenieria dei Sistemi (DIBRIS), Università di Genova, Via Opera Pia 13, 16145 Genova, Italy

**Keywords:** cyanoacrylate, caffeic acid, polypropylene carbonate, UV-blocking

## Abstract

Applications of cyanoacrylate monomers are generally limited to adhesives/glues (instant or superglues) and forensic sciences. They tend to polymerize rapidly into rigid structures when exposed to trace amounts of moisture. Transforming cyanoacrylate monomers into transparent polymeric films or coatings can open up several new applications, as they are biocompatible, biodegradable and have surgical uses. Like other acrylics, cyanoacrylate polymers are glassy and rigid. To circumvent this, we prepared transparent cyanoacrylate films by solvent casting from a readily biodegrade solvent, cyclopentanone. To improve the ductility of the films, poly(propylene carbonate) (PPC) biopolymer was used as an additive (maximum 5 wt.%) while maintaining transparency. Additionally, ductile films were functionalized with caffeic acid (maximum 2 wt.%), with no loss of transparency while establishing highly effective double functionality, i.e., antioxidant effect and effective UV-absorbing capability. Less than 25 mg antioxidant caffeic acid release per gram film was achieved within a 24-h period, conforming to food safety regulations. Within 2 h, films achieved 100% radical inhibition levels. Films displayed zero UVC (100–280 nm) and UVB (280–315 nm), and ~15% UVA (315–400 nm) radiation transmittance comparable to advanced sunscreen materials containing ZnO nanoparticles or quantum dots. Transparent films also exhibited promising water vapor and oxygen barrier properties, outperforming low-density polyethylene (LPDE) films. Several potential applications can be envisioned such as films for fatty food preservation, biofilms for sun screening, and biomedical films for free-radical inhibition.

## 1. Introduction

Cyanoacrylate monomers are well-known polymer precursors that are commonly used as super glue and as surgical wound closure adhesives. Cyanoacrylate monomers instantly polymerize by solidifying rapidly in the presence of moisture and this property forms the basis for designing super glues and surgical adhesives [1]. Some recent works also demonstrated that cyanoacrylates can be polymerized in solution in a controlled way to form transparent acrylic films or nanofiber mats [2]. Moreover, cyanoacrylate monomers were used as solvents for other acrylate polymers such as poly(methyl methacrylate) or PMMA [3]. Recent reports demonstrated that transparent poly(ethyl cyanoacrylate) (PECA) films can be cast from ecofriendly solvents [4]. These films were found to have unique slippery wetting characteristics as well as tribological properties while maintaining their biocompatibility [2,4]. The high biocompatibility of PECA has promoted its use as a drug-delivery system [5,6,7]. Although transparent PECA films have certain advantages over rapidly cross-linked porous networks, they are intrinsically brittle like many acrylic polymers [4]. The characteristic brittleness of acrylates is generally overcome by incorporating plasticizers in the form of small molecules such as phthalates, glycols, and certain citrates [7,8,9]. However, some plasticizers pose problems, related to food contact and biomedical–cosmetic applications, like increasing water uptake of the films, evaporation over time, leaching into objects of contact, etc. [10]. To circumvent this, one possible approach could be to compound PECA with other rubbery biopolymers without sacrificing its transparency. In this study, we demonstrate this concept by compounding PECA with poly(propylene carbonate) (PPC) in solution.

Poly(propylene carbonate) (PPC) is derived from carbon dioxide and is a biodegradable, transparent polymer which is generally used as a plasticizer in different polymers to enhance elastic properties due to its large elongation at break value [11,12,13,14] The addition of PPC into PECA matrix could improve processability, impact resistance, ductility and flexibility of the pristine cyanoacrylate. To best of our knowledge, incorporation of PPC into PECA polymer has not been demonstrated to date. Even though PECA and PPC are immiscible polymers, we observed that PPC can be blended into PECA up to 5.0 wt.% while maintaining transparency. In fact, lower concentrations of PPC (i.e., 2.5 wt.%) were shown to be sufficient to increase ductility of PECA up to 10 times. Moreover, we show that films exhibited a strong radical scavenging activity by adding a natural antioxidant molecule known as caffeic acid (CA). Caffeic acid is a phenolic acid that belongs to hydroxycinnamic acid family that can work in the prevention of several diseases, (cancer, coronary heart disease, diabetes, etc.) [15,16]. The capability of natural phenolic acids in preventing damaging oxidation reactions on foods, due to their antioxidant activity, has increased their use in food-packaging materials [17]. For example, caffeic acid molecules were grafted to chitosan for enhancing the antioxidant activity, up to 80% higher than neat chitosan films [18]. Caffeic acid also showed to strongly reduce the lipid oxidation of fish oil emulsion when incorporated into chitosan/cellulose composites [19]. The scavenging capacity of caffeic acid was found to be better than other polyphenols, like *p*-coumaric acid when added into chitosan-based films, or tyrosol in pectin biobased films [20,21]. Moreover, the films can act as protective ultraviolet (UV) absorbers with less than 0% transmittance within UVB (280–315 nm) and UVC (100–280 nm) and about 15% in UVA (315–400 nm) ranges. The development of UV-blocking properties is important to reduce food degradation for radical oxidation, such as the lipid oxidation [22]. The incorporation of natural antioxidants for providing antioxidant and UV-blocking properties has been also observed in Zechang et al. [23], who bonded lignin to cellulose nanocrystals (CNC) to create a biodegradable and active food-packaging material. Natural antioxidants such as lignin or modified lignin [24] are very effective antioxidants but the main drawback of UV-blocking packaging-materials based on natural compounds is that they usually have high opacity and strong color, which can hinder the perception of the product inside, such as lignin-based materials [25], and curcumin [26]. However, in this work, we developed transparent and UV-blocking films enabled by the incorporation of the natural polyphenol—caffeic acid. Moreover, the blend films also displayed promising water vapor permeation and oxygen barrier properties comparable and better than common food packaging films such as low-density polyethylene (LPDE). This unique combination of properties can increase applications of PECA polymers in areas such as cosmetics, biomedical and food packaging technologies. 

## 2. Materials and Methods

### 2.1. Materials

Ethyl 2-cyanoacrylate monomer (ECA, Permabond 101), poly(propylene carbonate) resin (typical average Mw 89,000–98,000 g/mol, 98% hydrolyzed), caffeic acid (≥98.0% high performance liquid chromatography (HPLC)) and cyclopentanone ReagentPlus^®^ (≥99%), were purchased from Sigma Aldrich ( St. Louis, MO, USA) and used as received. Deionized water was obtained from Milli-Q Advantage A10 Ultrapure Water Purification device (Merck, Darmstadt, Germany). DPPH (2,2-diphenyl-1-picrylhydrazyl) was purchased from Alfa Aesar (Kandel, Germany) and ethanol (≤100%) was purchased from Merck and both were used as received.

### 2.2. Preparation of the Films

**Figure 1:**
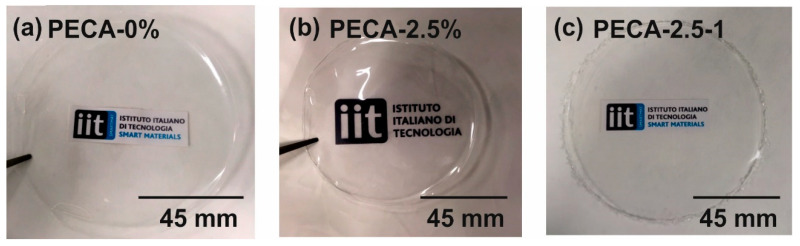
Photographs of (**a**) transparent pristine poly(ethyl cyanoacrylate) (PECA), (**b**) PECA blended with 2.5 wt.% of poly(propylene carbonate) (PPC) and (**c**) PECA blended with 2.5 wt.% of PPC with 1.0 wt.% of caffeic acid.

The films were made by polymerizing ECA monomer in the presence of PPC and caffeic acid, all dispersed in cyclopentanone. The slow evaporation rate of cyclopentanone (boiling point ~130 °C) leads ECA to form transparent PECA films [2,4]. Two PPC concentrations were studied, namely 2.5 wt.% and 5.0 wt.%, with respect to PECA (on dry basis). The films were labeled as PECA-2.5 and PECA-5, respectively. Antioxidant films contained 1.0 wt.% and 2.0 wt.% caffeic acid with respect to PECA on dry basis and were labeled as PECA-2.5-1 and PECA-5-2, respectively. As an example, proper quantities of caffeic acid and PPC were first dispersed in cyclopentanone and after their dissolution ECA monomer was added dropwise until 13.0 wt.% of ECA in solution was achieved. Solutions were mixed with a vortex mixer and sonicated in a water bath for 90 min at room temperature and with a frequency of 59 kHz. Subsequently, solutions were poured into a glass petri dish molds (90 mm diameter). After drying overnight under chemical hood at room temperature, transparent films easily peeled off the molds. Photographs of some representative films are shown in Figure 1. The concentrations of caffeic acid and PPC in the studied samples are summarized in Table 1. 

Note that we produced other potential combinations but the samples shown in Table 1 were selected among several other such combinations including films like PECA-5-1 or PECA-2.5-2. The selection was based on film homogeneity after drying such as the absence of macroscopic phase separation domains, and adequate mechanical properties like better ductility compared to PECA alone. 

### 2.3. Attenuated Total Reflection Fourier Transform Infrared (ATR-FTIR) Spectroscopy 

Infrared spectra were obtained with a single-reflection attenuated total reflection (ATR) accessory (MIRacle ATR, PIKE Technologies, Madison, MI, USA) coupled to a Fourier Transform Infrared (FTIR) spectrometer (Vertex 70V, Bruker, Ettlingen, Germany). All spectra were recorded in the 3800 to 600 cm^−1^ wavenumber range with a resolution of 4 cm^−1^, accumulating 128 scans to reduce the spectral noise. All data were analyzed with OPUS software.

### 2.4. Scanning Electron Microscopy (SEM), Atomic Force Microscopy (AFM), and Optical Profilometry 

Surface and cross-section morphologies of solvent cast films were characterized by scanning electron microscopy (SEM), using a JEOL JSM-6490LA microscope working in high vacuum mode, with an acceleration voltage of 10 kV. For the cross-section analysis, samples were immersed in liquid nitrogen for few seconds for fracturing. Before the analysis, samples were mounted on conductive stubs and coated with 10 nm of gold using Cressington sputter coater 208 HR. The surface topography of the films was studied using atomic-force microscopy (AFM) system E-100 (Park systems, Suwon, South Korea), with high-resolution imaging; scan size 50 × 50 µm. The size distribution and the number of voids and PPC-domains of the films obtained from several different SEM images have been estimated by using ImageJ commercial software. In order to characterize the microscale PPC rubbery domains within the bulk of the PECA films, PPC was etched by immersing the films in acetone for two days [11]. Acetone immersion tests with pure PECA showed no dissolution of the polymer in acetone during the immersion tests. 

### 2.5. Light Transmittance Analysis

The optical transmittance of films was determined using a Varian CARY 6000i Scan UV-Vis spectrophotometer (Walnut Creek, CA, USA) over the wavelength range from 200 to 800 nm. For the measurements, the freestanding films with a thickness of around ~80 µm were placed in a compartment with a light pathway of 2 cm × 2 cm. An empty test cell was used as a reference. 

### 2.6. Mechanical Characterization

Mechanical properties of the films were measured by uniaxial tensile tests on a dual column Instron 3365 universal testing machine (High Wycombe, England). Dog-bone shaped samples had a gauge length of 25 mm and a width of 4 mm. Strain displacement was applied with a rate of 10 mm/min. Stress–strain curves were recorded at 25 °C and 44 % relative humidity (RH). At least ten measurements were conducted for each sample and results were averaged to obtain a mean value. The stiffness of the material was defined by the Young’s modulus value, calculated from the slope of the linear part of stress–strain curve (before elastic limit). Both Young’s (elastic) modulus and elongation at break values were determined using the built-in software of the tensile tester.

### 2.7. Water Uptake and Film Hydrophobicity

For water uptake measurements, samples were first dried by conditioning them in a desiccator until no change in sample weight was measured. Dry samples were weighed on a sensitive electronic balance, and then placed in a 100% relativity humidity (RH) chamber at 25 °C. Once the equilibrium was reached, each sample was again weighed and the amount of adsorbed water was calculated as the difference between the final and the initial dry weight. Three measurements were taken and the results were averaged to obtain a mean value. Percent of water uptake was calculated as:

(1) $$\mathrm{Water}\;\mathrm{uptake}\;\left(\%\right)=\frac{m_{f}-m_{0}}{m_{0}}\;\times \;100$$

where $m_{f}$ is the sample weight at 100% RH and $m_{0}$ is the sample weight at 0% RH. Static water contact angle measurements were performed by using the sessile drop method using a Dataphysics 0CAH 200 contract angle goniometer equipped with a charge-coupled device (CCD) camera and image processing software operating under laboratory conditions (temperature 22–25 °C and relative humidity 50–60%). For the characterization, droplets of 5 μL volume MilliQ water were used. Up to 10-contact angle measurements were carried out on every sample at random locations and their average values are reported.

### 2.8. Oxygen and Water Vapor Barrier Measurements

Water vapor permeability (WVP) of all samples was determined at 20 °C under 100% relative humidity (ΔH %). As a desiccant anhydrous calcium chloride (CaCl_2_) was placed inside the test permeation cell (7 mm inside diameter, 10 mm inner depth), maintaining 0% RH inside the permeation cell. Films were cut into circles and mounted on top of the permeation cell. The permeation cells were placed in a 100% RH using deionized water container. The water vapor transferred through the film was determined from the weight change of the permeation cell every hour over a 7 h-period using an electronic analytical balance, Sartorius CPA225D (0.01 mg accuracy). The weight gain of the films was then plotted as a function of time. The slope of each line was calculated by linear regression and the water vapor transmission rate (WVTR) was determined as below:

(2) $$\mathrm{WVTR}\;\left(\frac{g}{m^{2}\cdot \mathrm{day}}\right)=\frac{\mathrm{Slope}}{\mathrm{Area}\;\mathrm{of}\;\mathrm{the}\;\mathrm{film}}$$

WVP measurements were conducted three times for each sample. The WVP value was calculated asmfollows:

(3) $$\mathrm{WVP}\;\left(\frac{g}{m\cdot \mathrm{day}\cdot \mathrm{Pa}}\right)=\frac{\mathrm{WVTR}\;x\;l\;x\;100}{p_{s}\;x\;\Delta \mathrm{RH}}$$

where l (m) is the film thickness measured with a micrometer with 0.001 mm accuracy, ΔRH (%) is the percent relative humidity gradient, and $p_{s}$ (Pa) is the saturation water vapor pressure at 25 °C (3168 Pa). The oxygen permeation tests of blend films were performed using an Oxysense 525oi device (Oxysense, Devens, MA, USA) equipped with a film permeation chamber. The system was operated according to American Society for Testing and Materials (ASTM) Method F3136-15. The tests were performed at standard laboratory conditions, i.e., 23 °C and 50% RH. The permeation chamber consisted of a cylinder divided into two parts (sensing well and driving well). The sensing well was equipped with a fluorescence sensor called oxydots, sensitive to the oxygen concentration. This chamber was purged with nitrogen while the other one (driving well) was kept open to ambient air. The films were cut into rectangular pieces (6 cm × 6 cm) and placed inside the chamber. The OxySense fiber-optic pen measured the oxygen concentration from the oxydots, at specific time intervals. Oxygen transmission rates (OTRs) of the blend films were measured by monitoring the oxygen uptake with time using Oxysense OTR software. At least 10 measurements were taken for each sample with a minimum R^2^ value of 0.95. 

### 2.9. Antioxidant Effect Measurements 

The migration of caffeic acid from the polymer films was measured by studying the specific migration of the antioxidant into the fatty food simulant by UV-spectroscopy, according to established liquid immersion protocols [20,27]. Caffeic acid is slightly soluble in water but highly soluble in ethanol, which is the main component of the fatty food simulant used for antioxidant effect measurements [20]. The method described by Lopez-de-Dicastillo et al. [27] was followed with some modifications. Films were cut into 3-milligram pieces and were immersed in 3 mL of pure ethanol. The concentration of caffeic acid released from film to the solution was analyzed by measuring the UV-absorption spectra of the liquid at certain time intervals. All measurements were repeated at least five times to ensure the reproducibility with Varian CARY 6000i Scan UV-Vis spectrophotometer (Walnut Creek, CA, USA). Prior to measurements, a calibration curve was constructed by correlating the absorbance intensity of caffeic acid at 327 nm in ethanol by using several caffeic acid–ethanol solutions (see Figure S1a,b in Supplementary Material). 

Results were represented in terms of milligrams of caffeic acid released per gram of original dry film. The antioxidant capacity of the films was measured in terms of scavenging activity of the caffeic acid released from the films against a free stable radical, 2,2-diphenyl-1-picrylhydrazyl radical (${\mathrm{DPPH}}^{\cdot }$). Similar to the previous experiment, 3 mg of films were immersed in 3 mL of (${\mathrm{DPPH}}^{\cdot }$) dissolved ethanol solutions as described in the literature [28]. The antioxidant effect of CA would induce a color change of the radical solution diminishing the absorption of the characteristic of at 517 nm as the release takes place. The antioxidant capacity was determined by collecting the UV-absorption spectra of the liquid at certain immersion time-intervals and the percent radical inhibition was calculated as:

(4) $$Radical\;Inhibition\;\left(\%\right)=\frac{A_{1}-A_{2}}{A_{1}}\;\times \;100$$

A_1_ is the absorbance of the sample solution with ${\mathrm{DPPH}}^{\cdot }$ 517 nm and A_2_ is the absorbance of the ${\mathrm{DPPH}}^{\cdot }$ control solution. All measurements were performed at least five times and with different samples to ensure reproducibility.

## 3. Results and Discussion

### 3.1. Morphological and Mechanical State of the Films 

**Figure 2:**
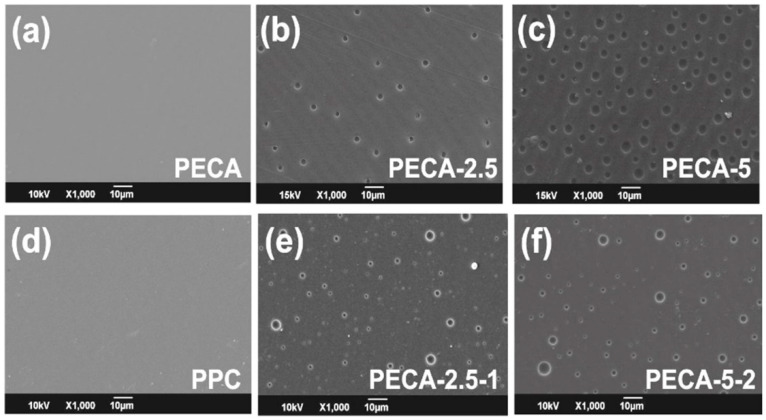
Surface scanning electron microscopy (SEM) images of (**a**) pure PECA, (**b**) PECA with 2.5 wt.% of PPC (PECA-2.5), (**c**) PECA with 5.0 wt.% of PPC (PECA-5), (**d**) pure PPC, (**e**) PECA-2.5 with 1.0 wt.% of caffeic acid, and (**f**) PECA-5 with 2wt. % of caffeic acid.

The surface morphology of samples has been analyzed by SEM measurements as shown in Figure 2. Pure PECA and pure PPC solvent-cast films had smooth and featureless morphologies as displayed in Figure 2a,d. Samples obtained from PECA containing 2.5 wt. % and 5.0 wt.% of PPC (PECA-2.5 and PECA-5) demonstrated void structures on their surfaces. The formation of these voids is related to the phase separation dynamics at the polymer-air interface during drying of the films. This concept is also known as structure formation due to polymer demixing in solvent-cast systems [29]. In fact, surface microstructure formation during drying of polymer blends has been very commonly observed and can even be controlled for patterning and soft lithography fabrication [30]. The surface structures due to phase separation at the interfaces can be very random, ordered or interpenetrating, depending on the polymer miscibility, polymer surface energy, solvents, drying conditions, and substrate effects. Walheim et al. [29] indicated that in certain polymer blends spherical surface voids form, if the common solvent is a better solvent for the higher surface energy (more hydrophilic) polymer. Moreover, the dispersion state of one polymer in the other is important during phase separation. Mono- or poly-dispersed polymer blends create different surface morphologies as they dry. This is also known as surface stratification and segregation of polymer-polymer mixtures [31]. In our system, as will be demonstrated later, PPC forms or segregates into spherical micro-domains within the PECA matrix. These domains also appear at the polymer-air interface and as the solvent evaporates away from the surface, the domains can slightly migrate into the bulk film leaving partial voids at the interface. 

**Figure 3:**
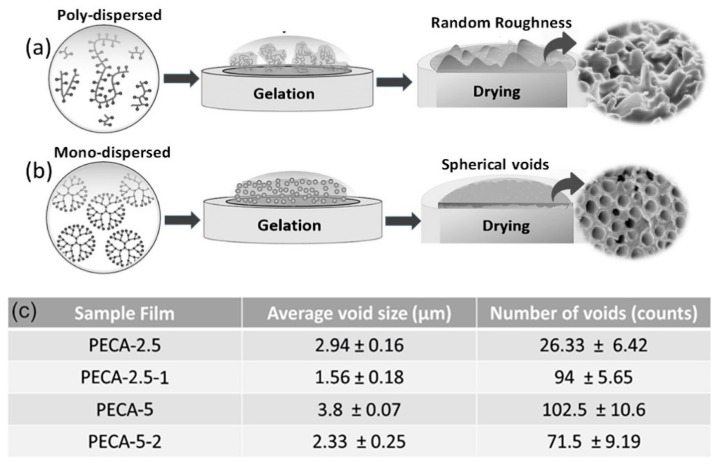
(**a**) Schematic description of poly-dispersed and (**b**) monodispersed polymer solution drying and formation of surface roughness features. (**c**) Tabulated average surface void size (diameter) and the number of surface voids of all PECA–PPC blends studied. All data are collected from a SEM viewing window of 132 × 90 microns and represented as mean ± SD (standard deviation) of three measurements.

As shown schematically in Figure 3a, drying of poly-dispersed polymer phases results in a random surface roughness whereas a monodispersed (micellar like) polymer phase drying causes spherical void-like features on the surface (Figure 3b). AFM measurements confirmed the formation of these voids and they are displayed in Figure S2 (Supplementary Material). Detailed image processing of several SEM images similar to the ones in Figure 2 indicated that on average, the void size of PECA-2.5 films (about 3-μm diameter) is smaller than PECA-5 films (about 4-μm diameter) (see Figure 3c). Adding the antioxidant, caffeic acid, decreased the average diameter of surface voids. In both cases, surface void sizes declined by approximately a factor of two. 

Another statistical parameter is the number of surface voids in the polymer blends. Figure 3c also lists the average number of voids counted on each film surface (132 × 90 μm). PECA-2.5 surface has the least number of voids among the samples. Adding caffeic acid, however, significantly increases the number of voids (approximately threefold) on the surface of PECA-2.5 films. This could be due to the interference of caffeic acid in the state of dispersion of rubbery polyphenylene oxide (PPO) within PECA. Possibly, under this specific blend conditions, caffeic acid can enhance the formation of more micellar rubber domains that resemble the effect of crystalline fillers on morphology development in elastomeric polymer blends [32]. The opposite effect was observed in PECA-5-2 blends in which 2 wt.% of caffeic acid decreased the number of voids by a factor of 1.3, possibly implying slightly better compatibility between the two immiscible polymers. Although the physicochemical reasons behind the changes in surface void size and numbers due to caffeic acid are beyond the scope of this work, it may be argued that the decrease in surface void size (diameter) and increase or decrease in their number could be related to the changes in the interfacial surface energy between the two polymers due to molecular interactions with caffeic acid [33]. As mentioned earlier, phase separation in the form of circular micro-domains was also observed in the bulk of the polymer blends. 

**Figure 4:**
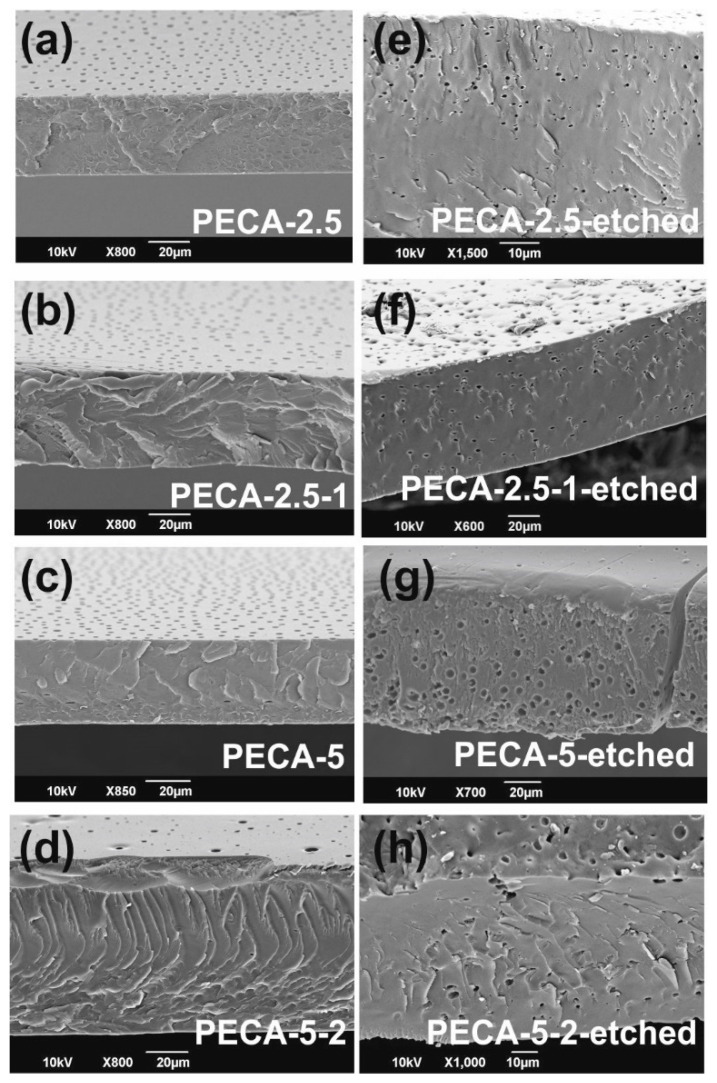
Cross-section SEM images of as-prepared (**a**–**d**) and solution etched (**e**–**h**) films. Acetone dissolved away the PPC leaving behind pores similar to the ones observed on the surfaces of the as-prepared films.

The bulk morphology of the blends was studied by analyzing the cross-section SEM images (Figure 4). Normally, SEM imaging of polymer blends does not detect any phase separation regions as polymers are insulators (Figure 4a–d), unless one of the polymers is selectively stained or solvent etched away for microscopic detection. In order to inspect PPC distribution across the thickness of the polymer films, they were immersed in acetone solutions (2 days) to swell and dissolve the PPC, as PECA does not dissolve in acetone. Due to the dissolution of PPC, several spherical PPC micro-domains appear throughout the cross section (Figure 4e–h), similar to the surface voids. Such micro-domains have also been observed in PPC-modified poly(lactic acid) (PLA) films [13,34]. In such a blend, Yao M. et al. [13] also demonstrated that maleic acid could be used to modulate the size and number of the rubbery PPC domains in PLA. Controlling the size and the number of rubber domains in rigid polymers is imperative for mechanical longevity in certain applications in plastic industry [35], since the rubber domain size or dimeter can vary orders of magnitude, when the blend concentrations are slightly changed. In our blends, however, there is no such large variation in the size of phase-separated micro-domains when PPC concertation is doubled besides adding caffeic acid does not destabilize the morphology of the blends. 

Both PPC and caffeic acid influenced the mechanical properties of PECA. Pristine PECA films had on average 1.4 GPa elastic modulus with a very low elongation at break value of 1.7%, typical of acrylics. Adding 2.5 or 5.0 wt.% PPC to PECA did not significantly influence the elastic modulus and in both cases the average modulus was found to be 1.3 GPa. Elongation at break values however increased to 17% and 3%, respectively. PECA-2.5 demonstrated much better elastic properties than PECA-5 and this could be due to the increased number of rubbery domains in PECA-5 with more interfacial adhesion failure sites that can cause elongation failure. This is also observed in rubber-modified rigid polymers and studies indicate that proper modification of interfacial adhesion diminishes early breaks during elongation [36]. Adding caffeic acid to PECA-2.5 (PECA2.5-1) did not affect the elastic modulus of the blend but elongation was reduced to about 10%, instead. In the case of PECA-5 films, caffeic acid (PECA-5-2) improved elongation levels to 7%, also without a significant impact on the elastic modulus of the blend. We can conclude that films having higher PPC content can attain acceptable levels of elasticity/flexibility with the addition of the proper amount of caffeic acid (see Figure S3, Supplementary Material).

### 3.2. Chemical Characterization

**Figure 5:**
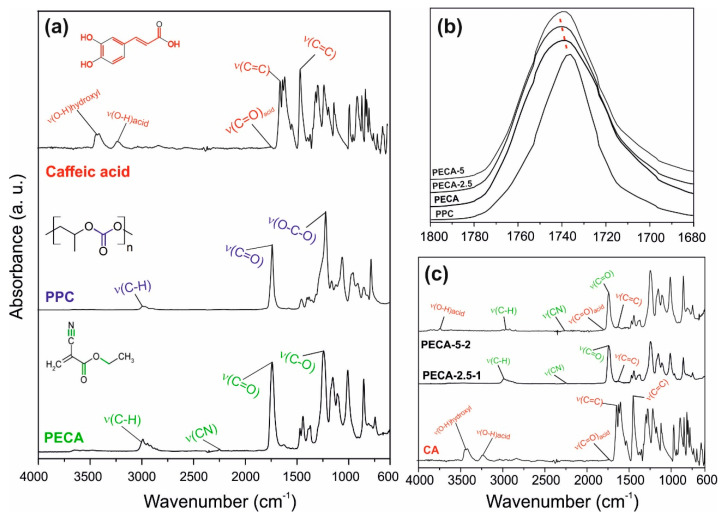
(**a**) The molecular structure and the infrared spectra of the pristine PECA, PPC, and caffeic acid covering the wavelength region 4000–600 cm^−1^. The characteristic peaks of PECA, PPC, and caffeic acid are indicated on each compound spectrum. (**b**) Stretching vibration band of ν(C=O) bonds belonged to pristine PECA, PPC, and PECA–PPC blends (PECA-2.5 and PECA-5). (**c**) The infrared spectra of PECA-2.5-1, PECA-5-2, and caffeic acid, with the characteristic peaks assigned, in the region 4000–600 cm^−1^.

Figure 5a shows the infrared spectra in the region 4000 to 600 cm^−1^, of the pristine PECA, PPC, and caffeic acid. The nitrile stretching vibration ν(C≡N) at 2230 cm^−1^, the carbonyl ν(C=O) stretching bond at 1742 cm^−1^, the double bond (C=C) at 1614 cm^−1^ and the vibration band of ν(C-O) at 1248 cm^−1^ were easily identified in pure PECA films [4]. The principal bonding bands of PPC were the ν(C-H) stretching band at 2995 cm^−1^, the vibration band associated to carbonate group ν(C=O) at 1739 cm^−1^ and the strong band with a shoulder at 1220 cm^−1^ related to ν(O-C-O) vibrations [37,38]. Caffeic acid spectra showed typical bands corresponding to common hydroxycinnamic acids [39]. The stretching vibration ν(O-H) associated with the carboxylic acid and hydroxyls groups of the benzene moiety is located in the range of 4000-2600 cm^−1^, while the ν(C=O) vibration band of α,β-unsaturated carboxylic acid is located at 1645 cm^−1^. The stretching vibrations of the alkene group ν(C=C) associated with the benzene group and acyclic double bond, conjugated in parallel with carboxylic acid produced vibration bands within1640-1445 cm^−1^ [40,41]. Figure 5b shows range specific (1800-1680 cm^−1^) infrared spectra of pristine PECA, PPC, and PECA–PPC blends with 2.5 and 5.0 wt.% of PPC (PECA-2.5 and PECA-5, respectively), where the stretching vibration of the carbonyl group is located at (1740 cm^−1^). A slight shift of carbonyl bond location towards higher wavenumbers indicates bond length decrease due to external influences like blending, rather than hydrogen-bonding interactions. Moreover, the absence of hydrogen donor groups in both PECA and PPC polymers can lead to partial chain entanglements via dipole–dipole interactions affecting ν(C=O) bonds and causing a slight shift towards higher wavenumbers in the case of PECA-2.5 films that can also lead to changes in elasticity of blends [41]. In the case of PECA-5, no such shift is noticed, indicating no hydrogen bonding interactions as well as no carbonyl bond length changes. It is known that in certain polymer blends where no hydrogen bonding interactions occur, bond vibrational/length changes can be attributed to polymer chain entanglements which depend on dispersion states, concentrations, and interfacial energy between immiscible polymers [42].

The infrared spectra of the antioxidant-loaded films are displayed in Figure 5c. The characteristic bands of PECA, like the nitrile stretching vibration ν(C≡N) at 2230 cm^−1^ and the stretching vibration at 1742 cm^−1^ from carbonyl functional group ν(C=O) and ν(C-O) at 1248 cm^−1^, are detected. Due to the low concentration of PPC polymer in the blends, the bands correlated with its functional groups are barely visible in the spectra. The stretching band of the aromatic ring due to caffeic acid, in the region 1680–1491 cm^−1^, as well as some bands related to the carboxylic groups, like ν(C=O) and ν(O-H), can be distinguished, especially in the PECA-5-2 sample. FTIR spectra of caffeic acid-added blends indicated no noticeable changes or shifts in the main vibration bands, likely due to the low concentration of the antioxidant. This could suggest that caffeic acid appears to be a physical filler rather than a chemically interacting compound in the blend. However, this does not necessitate the exclusion of its potential role in surface energy modulation or dispersion state of polymers in solution or during film drying. 

### 3.3. UV-Blocking Characteristics

**Figure 6:**
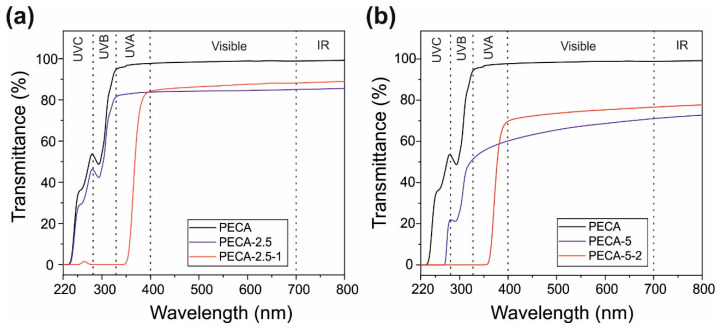
(**a**) The transmittance spectra of pristine PECA (in black), PECA blends in 2.5% wt. of PPC (PECA-2.5) and caffeic acid added in 1.0 wt.% (PECA-2.5-1). (**b**) PECA blends in 5.0% wt. of PPC (PECA-5) and caffeic acid added in 2% wt. (PECA-5-2). The PECA–PPC blends are shown in blue while the blends with caffeic acid are in red.

The ultraviolet radiation from the sun light is divided in the following wavelength ranges: UVC (220−280 nm), UVB (280−320 nm), and UVA (320−400 nm). UVC and UVB radiation ranges are the most important ones to block as they can cause photo-oxidation, photo-carcinogenesis and photo-aging [43,44]. The absorption of many acrylic polymers diminishes rapidly between 220–400 nm, becoming transparent to UVB and UVA radiation wavelengths [45]. The transmittance spectra of the PECA films modified with PPC, with and without caffeic acid, are displayed in Figure 6. Transmittance is the percentage of radiation that passes through a material (percent transmission), and for UV blocking materials it should be close to zero. This is generally difficult to achieve in polymer films transparent to the visible range, unless specific nanoparticles or quantum dots are dispersed in the films [46]. 

Regarding the visible range of the electromagnetic spectrum, although PPC-modified PECA films do not feature 100% transparency due to scattering by the dispersed PPC micro-domains shown above, their visible light transmittance between 75 and 80% can be considered rather satisfactory for applications such as transparent bio-based packaging materials (see Figure 1). For instance, rubber modified synthetic acrylic polymers, such as poly(methyl methacrylate) and ethylene–vinyl acetate copolymer blends that were developed for packaging also feature similar visible spectrum transparency values around 80–85% [47]. In general, transparency of immiscible biopolymer blends, particularly PLA blends, is very difficult to attain unless certain methods are applied such as reactive compatibilization [48]. The visible light transparency of PECA films containing 5.0 wt.% PPC is quite poor, with about 60% transmission levels, as seen in Figure 6b. Adding 2 wt.% caffeic acid in this blend however, improves visible light transparency close to 70% level. As explained in Maruashi et al. [49], the transparency of an immiscible blend depends on the size and distribution of the spherical micro-domains (island–sea structure) in the polymer bulk, which is closely related to the compatibility. Referring to the observations discussed related to Figure 2 and Figure 3, caffeic acid appears to be reducing surface and bulk void sizes. In the case of PECA-5-2, about a 1.3-times reduction in number of voids was also measured. The resulting optical effect in the visible range is reflected as amplified transparency. 

Regarding the UV range of the electromagnetic spectrum, Figure 6a shows that pure PECA films have moderate to low absorption. In fact, cyanoacrylates are known as partial UV light absorbers and by modulating their monomeric structures, forensic scientists have developed advanced fingerprinting and DNA isolation methods under UV illumination [50]. The transmittance of pure PECA films ranges from 0 to 55% within UVC, from 55 to 90% within UVB and remains constant at 90% within UVA range. PPC, on the other hand, transmits more than 85% of all UV radiation (not shown here for brevity) and is prone to rapid photo-induced degradation [51]. As seen in Figure 5a, compounding 2.5% PPC in PECA causes just a slight reduction in UV transmission. The films with 5.0 % of PPC, on the other hand, show a more pronounced reduction of the UV transmission, Figure 5b. In particular, the transmission in UVC becomes practically zero, within UVB, it ranges from 20% to 50%, and within UVA it hardly reaches 60%. Adding caffeic acid to PPC–PECA films completely blocks the transmission of UVC and UVB wavelengths and the films start to transmit light only above 350 nm. The UVA range is further classified as UVA2 for rays between 320 and 340 nm, and as UVA1 for rays between 340 and 400 nm. Zero transmission within the UVA1 range is the most challenging for sun screening applications and the state-of-the-art materials rely on ZnO as the absorber for both UVA1 and UVA2 wavelength ranges [52,53]. In the case of PECA–PPC films, protection against UVA1 range is best obtained when caffeic acid concentration is maintained at 2.0 wt.%. For instance, in Figure 6b, PECA-5-2 demonstrates about 17% transmission at 375 nm compared to PECA-2.5-1 that transmits about 35% at the same wavelength. Table 2 shows comparisons among published works on UV blocking capabilities of various transparent polymer films including the present study. It appears that achieving UV blocking without using nanoparticles such as ZnO or TiO_2_ is rather challenging and only a few all-organic compatible systems exist such as furan-based polyesters or modified dopamine-containing polymers [54,55].

### 3.4. Hydrophobicity and Water Vapor Absorption

Pure PECA films were hydrophobic with an average static water contact angle of about 100^°^. Adding PPC and caffeic acid did not alter their hydrophobicity and static water contact angles remained similar in all blends studied. Percent of water vapor uptake of the films (at 100% RH) is displayed in Table 3. Within the experimental measurement uncertainty, adding PPC to PECA does not seem to have a significant effect on the water uptake behavior of pure PECA. Owing to the hygroscopic nature of caffeic acid [60], the water uptake values of all blends with caffeic acid were almost doubled depending on the caffeic acid concentration, as shown in Table 3. In particular, PPC is known to have a low water uptake or water vapor sorption value of about 2% at 100 RH% and pure PECA shows water vapor absorption values of about 3–4% at 100% RH. Water uptake values of caffeic acid-containing blends, although increased with respect to the individual polymers and blends, they remain below 10% at 100% RH. These values are comparable to common biopolyester-thermoplastic starch blends (some of which are already commercialized) and may be acceptable for packaging applications [61].

### 3.5. Water Vapor Barrier and Oxygen Gas Permeation

**Figure 7:**
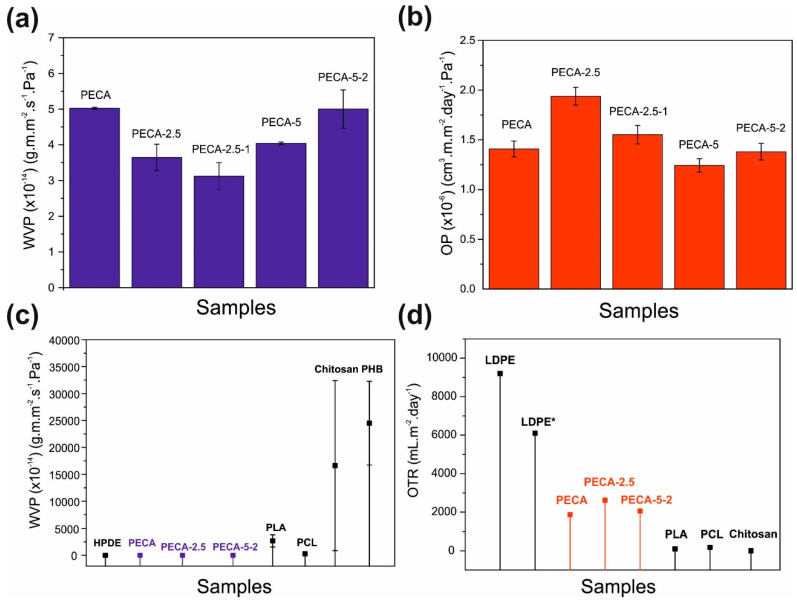
(**a**) Water vapor permeability and (**b**) oxygen permeability results of all films. All data represent the mean ± SME of three measurements. (**c**) Comparison of water vapor permeability (WVP) and (**d**) oxygen transmission rate with other polymers: high-density polyethyelene (HDPE) [64], poly(lactic acid) (PLA) [67,68], polycaprolactone (PCL) [66,69], chitosan [42,66], polyhydroxybutyrate (PHB), low-density polyethylene (LDPE), LDPE with 1% wt. of linanool (LDPE*) [64].

Figure 7a represents the water vapor permeability (WVP) of pure PECA and the PECA–PPC blends normalized by the film thickness. Results of Figure 7a indicate that compounding PPC enhanced the water vapor barrier properties diminishing the water permeability of pristine PECA by half in the case of 2.5 wt.% and by 1.5 times in the case of 5.0 wt.% PPC. This reduction was justified by the intercalation of PPC into the semi-crystalline polymer matrix that could likely reduce the chain mobility, leading to a higher tortuosity pathway of the water vapor molecules, as observed in other polymeric blends [62,63]. Adding caffeic acid by 1 wt.% in PECA-2.5 does not alter the reduced WVP properties due to PPC, however, adding 2.0 wt.% caffeic acid in PECA-5 slightly increased the WVP in the blend. Nonetheless, WVP of PECA-5-2 blend does not exceed the value for pure PECA as seen in Figure 7a. This appears to be in agreement with the water uptake results, since the hygroscopic character of caffeic acid is known to promote water permeation at high relative humidity conditions [60]. Similarly, the oxygen gas permeability (OP) of pristine PECA, and all the blends were measured and the results are shown in Figure 7b. Incorporating 2.5 wt.% PPC into pure PECA slightly increased (1.4 times) oxygen permeability but adding 1.0 wt.% caffeic acid to this blend (PECA-2.5-1) compensated this increase, lowering the values to the levels of pure PECA. Adding 5.0 wt.% PPC to PECA appears to be more advantageous compared to 2.5 wt.% since it practically does not increase the OP of pure PECA (with even a slight decrease). Moreover, in this case, adding caffeic acid in PECA-5 films did not cause any significant changes in the OP values compared to pure PECA, considering the measurement uncertainty levels (Figure 7b). It may be argued that the OP levels of the blend films are controlled by the physical blending effects of the components rather than chemical oxygen quenching/scavenging capability of caffeic acid. Physical blending can either diminish or enhance OP values depending on the diffusion paths generated within the polymeric networks [12]. In the present case, caffeic acid appears to be compensating for blends that appear to enable easier oxygen diffusion like the films PECA-2.5. Most probably, this effect is due to well-known singlet oxygen quenching properties of caffeic acid [22,64]. The fact that compounding 5.0 wt.% PPC and 2.0 wt.% caffeic acid in pure PECA did not deteriorate OP performance of pure PECA is encouraging considering other benefits such as UV-blocking properties and the ductility of the blend. Inspecting Figure 7c and d also reveal that both pure PECA and PECA/PPC blends with or without caffeic acid display competitive WVP values compared to conventional polymers such as HDPE, PCL, and PLA. Moreover, OTR values of the blends are superior to common food packaging LDPE films (including improved LDPE films with antioxidants) and reasonably close to PCL, PLA, and chitosan biopolymers [65,66,67,68,69,70]. As such, these films can be implemented in food packaging applications either as single-layer films or in combination with other polymers in the future.

### 3.6. Antioxidant Release and Capacity

**Figure 8:**
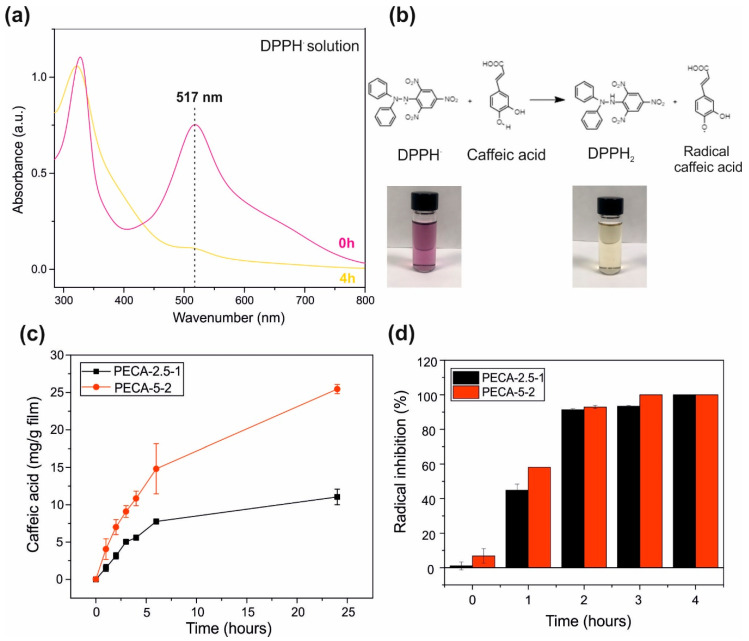
(**a**) The UV-Vis spectra of the radical solution (${\mathrm{DPPH}}^{\cdot }$), at time point zero (in violet) and after 4 h (in yellow). (**b**) The mechanism of the scavenging reaction between the caffeic acid and (${\mathrm{DPPH}}^{\cdot }$ ). The color change of the solution after scavenging reaction, from strong violet to yellow, is also represented. (**c**) Transient caffeic acid release profiles for PECA-2.5-1 and PECA-5-2 in (mg/g film). (**d**) Percent radical inhibition values of PECA-2.5-1 and PECA-5-2 films over a 4-h period. All results are represented as mean ± SD of three measurements.

The antioxidant capacity of caffeic acid was measured against the radical ${\mathrm{DPPH}}^{\cdot }$ using spectrophotometric methods [70]. The release of the antioxidant caffeic acid into the radical solution will lead to a decrease in the characteristic absorption band of the radical at 517 nm with time, as shown in Figure 8a. The first step of the scavenging mechanism of the caffeic acid against ${\mathrm{DPPH}}^{\cdot }$ was proposed in Li et al. [71] is represented in Figure 8b. Briefly, caffeic acid donates the hydrogen atom ($H^{\cdot }$) to the radical ${\mathrm{DPPH}}^{\cdot }$ resulting in a stable DPPH_2_ form. This reaction will lead to the color change of the violet radical ${\mathrm{DPPH}}^{\cdot }$ solution into yellow as it reacts with the antioxidant caffeic acid [70]. Most commonly, the antioxidant effectiveness of films developed for preserving food against oxidation is measured by immersing the samples in a food simulant and monitoring the migration rate of the antioxidant at certain time intervals. Ethanol is used as the main release medium, as fatty food simulant, which indicates the aptitude of the embedded antioxidants against foods with high lipid contents. The solubility of caffeic acid in ethanol facilitates precise measurement of antioxidant release kinetics and establishing correlations related to sustained release and fatty food preservation performance of the films. The amount of antioxidant released was expressed as mg caffeic acid/g film and is shown in Figure 8a. For the same reason, the antioxidant capacity of these extracts as percent radical inhibition was calculated and plotted in Figure 8b. Note that no burst release of significant amounts of the antioxidant from the films is observed indicating that the developed films have controlled release capability [72]. After 24-h period, PECA-2.5-1 released about 11 mg/g film of antioxidant and PECA-5-2 released 25 mg/g film. These quantities (11 and 25 mg/g film/day) appear to be adequate enough and agree with earlier reports on other antioxidant release data to ensure the quality of foodstuffs as well as release dosage of antioxidant drugs in the human body. Note that caffeic acid is a natural phenolic compound present in our daily diet, for example, typical brewed coffee consumption results in 250–500 mg of caffeic acid intake per day, ten times the concentration released during the tests [73]. Despite the low concentration of caffeic acid present in the films, 100% radical inhibition was achieved after 2 h for both films containing 3 and 7 mg/g film of caffeic acid present in PECA-2.5-1 and PECA-5-2 films, respectively. This shows promising antioxidant capability of the developed composite films that may be useful in preserving foodstuffs rich in lipids, such as meats and oils, even though more work needs to be conducted for food preservation applications. The 100% radical inhibition was obtained after 4 h in gelatin-based films with caffeic acid incorporated through cross-linking, showing also an excellent capacity of preventing the lipid oxidation in fresh pork samples [74]. The grafting of caffeic acid in propylene films led to a radical inhibition of 89 ± 6% after 30 min, besides preventing the oxidation of the bioactive compounds present in orange juice [75].

## 4. Conclusions

Transparent, UV-blocking and antioxidant PECA–PPC polymer blend films containing caffeic acid were fabricated from biodegradable polymers. The films demonstrated multifunctional properties such as good UV-blocking capability (UVA–UVC range), antioxidant effect and good water vapor and oxygen barrier properties. Films were colorless and highly transparent, contrary to the characteristic colorful materials obtained from natural antioxidants such as curcumin.

The incorporation of PPC into PECA polymer improved the ductility of the acrylic as well as diminishing its water vapor transport. Caffeic acid could be released into food simulants and acted as an effective antioxidant. No burst release of caffeic acid from the films substantiated that the blend films can effectively encapsulate hydrophilic agents or drugs and enable their sustained release over extended periods. These new films can be quite suitable for certain medical/cosmetic applications even as packaging films for preserving oxidation and UV sensitive biomaterials.

## Figures and Tables

**Table 1:** Composition of the dry films studied in this work. CA stands for caffeic acid, PPC for poly(propylene carbonate) and PECA for poly(ethyl cyanoacrylate).

Polymer Film	Concentration of PPC (wt.%)	Concentration of CA (wt.%)
PECA	-	-
PECA-2.5	2.5	-
PECA-2.5-1	2.5	1.0
PECA-5	5.0	-
PECA-5-2	5.0	2.0

**Table 2:** UV blocking properties of different polymeric systems reported in literature. They are compared against the best performing sample from this study. Abbreviations, PVA: Polyvinyl alcohol; POSS: Polyhedral oligomeric silsesquioxanes; PMMA: Polymethyl methacrylate.

Polymer	Additive	% Transmittance			Comments	Reference
		UVC	UVB	UVA		
**PVA**	Dopamine−melanin Nanoparticles (NP)	0	0	10–70%	Best performance: 5 vol.% NP	[44]
**PVA**	Carbon quantum dots	0	10%	5–60%	Best performance: 1 wt.% coating on PET	[46]
**PMMA**	ZnO quantum dots	0	0	40–50%	Best performance: 0.05 wt.% ZnO	[53]
**PMMA**	ZnO quantum dots	0	0	60%	Best performance: 2.4 wt.% ZnO	[53]
**Waterborne acrylic**	TiO_2_–Al_2_O_3_–POSS	0	0	60%	Best performance: POSS	[54]
**Bifuran polyester**	Furan-Based Dicarboxylic Acids	0	0	5%	Furan blocks UV	[55]
**PVA**	Wood nanofibers	0	5–10%	80%	Best Performance: 10 wt.% sulfated nanofibers	[56]
**Fish Gelatin**	ZnO nanorods	0	0	3–15%	Best Performance: 5 wt.% ZnO	[57]
**Carrageenan**	ZnO nanoparticles	5%	5%	n/a	Best Performance: 5 wt.% ZnO	[58]
**Sunscreen cream**	Lignin	15–45%	20–60%	25–80%	Best Performance: 10 wt.% Lignin	[59]
**PPC**	TiO_2_/lignin	7–70%	13–70%	13–70%	Best Performance: 5 wt.% lignin-TiO_2_	[51]
**PECA–PPC**	Caffeic acid	0	0	17%	Best Performance: 2% caffeic acid	This study

**Table 3:** Water uptake values of all PECA–PPC blends under 100% of relative humidity (RH). All results were expressed as mean ± SME of three experiments.

Sample Film	Water Uptake (%)
**PECA**	4.17 ± 0.89
**PPC**	2.6 ± 0.3
**PECA-2.5**	3.02 ± 0.38
**PECA-2.5-1**	6.95 ± 0.49
**PECA-5**	4.31 ± 0.40
**PECA-5-2**	8.78 ± 0.76

## Supplementary Materials

The following are available online at https://www.mdpi.com/2073-4360/12/9/2011/s1.
