# Supplementary material for: UV-Blocking, Transparent, and Antioxidant Polycyanoacrylate Films
Source: Polymers (Basel). 2020 Sep 3;12(9):2011. doi: 10.3390/polym12092011 (PMC7564323; doi:10.3390/polym12092011)
Supplement: Supplementary file 1 [file polymers-12-02011-s001.pdf]

Supplementary Material

UV-Blocking, Transparent and Antioxidant Polycyanoacrylate Films

Ana Isabel Quilez-Molina[1,2,*], *Lara Marini*[1], *Athanassia Athanassiou*[1], *Ilker S. Bayer*[1] .

[1] Smart Materials, Istituto Italiano di Tecnologia, Genova, 16163 Italy
[2] DIBRIS, Università di Genova, Via Opera Pia 13, 16145, Italy

***** Correspondence: ana.quilez@iit.it; ilker.bayer@iit.it

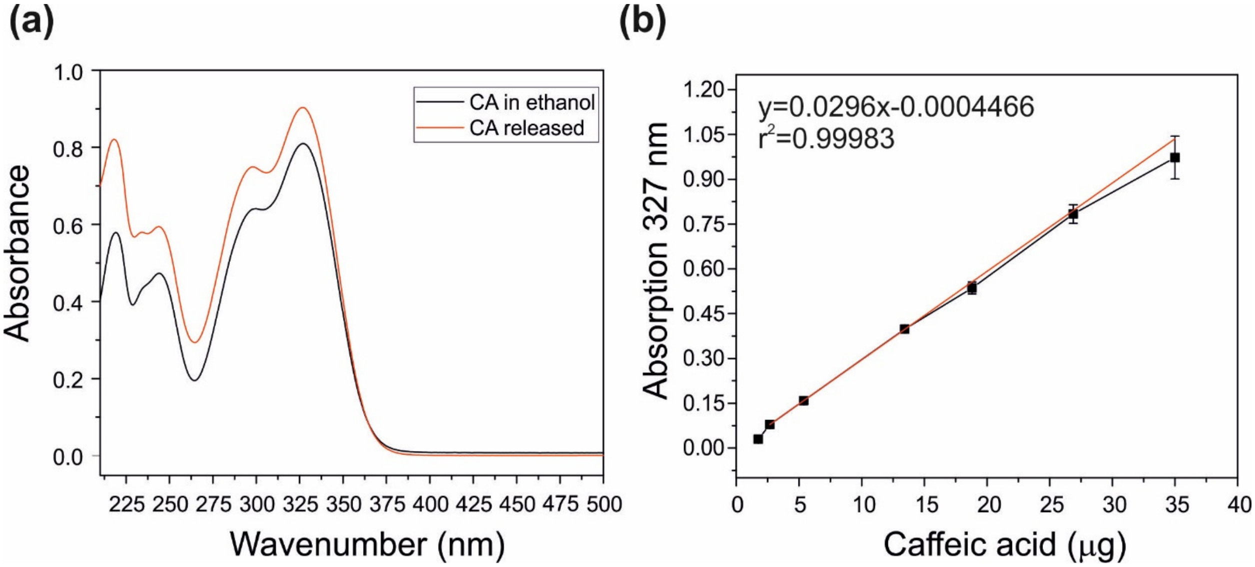

Fig. S1. (a) The UV-spectra of caffeic acid dissolved in ethanol (black) was used to calculated the calibration curve **(b)**. In **(a)** also the UV-spectra of the caffeic acid released from the PECA-5-2 after a time-interval was also presented in red.

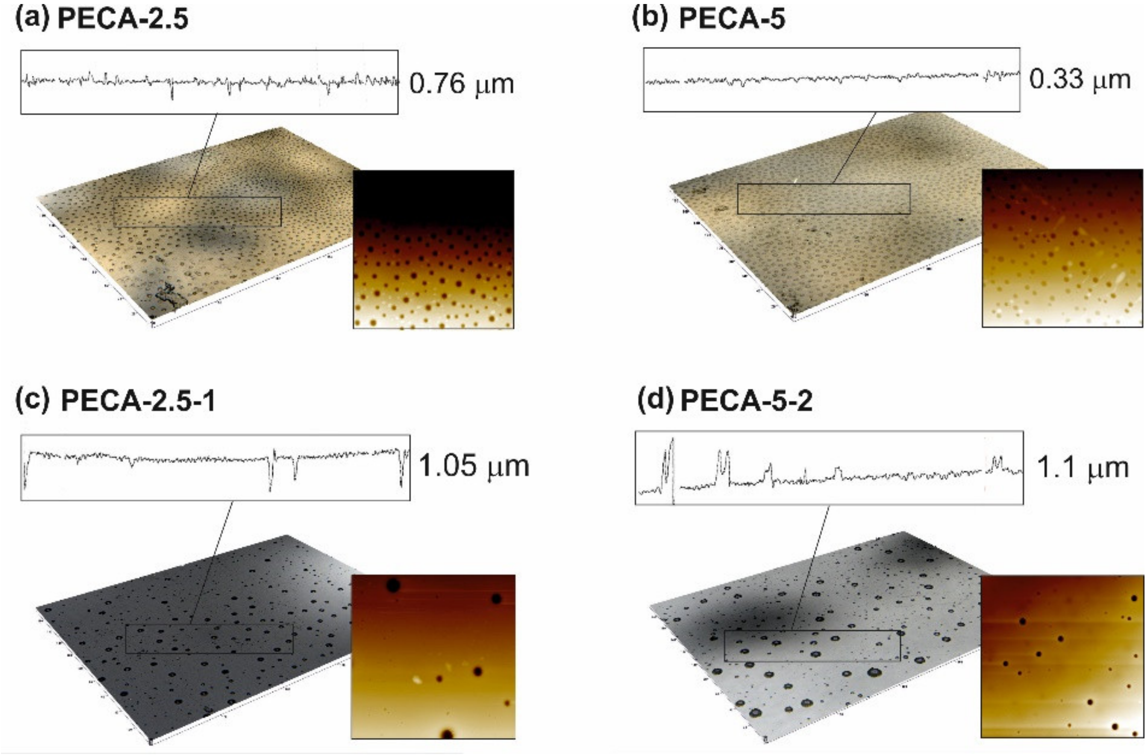

Figure S2. In the right side of the images were inset the topography study with the roughness profile and depth of pores, while in the left side the AFM photograph of samples were shown. PECA with 2.5 and 5% wt of PPC, are labeled as PECA-2.5 **(a)** and PECA-5 **(b)**, respectively. While PECA-2.5 with 1% wt. of caffeic acid and PECA-5 with 2% wt. of caffeic acid, are represented as PECA-2.5-1 **(c)** and PECA-5-2 **(d)**, respectively.

(a)

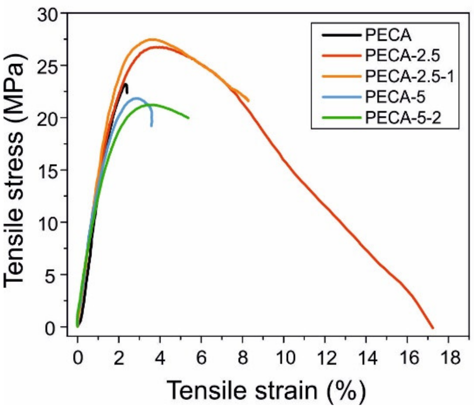

(b)

Sample labeled	Young's Modulus (MPa)	Elongation (%)
PECA	1344 ± 268	1.67 ± 0.68
PPC	22.05 ± 4.1	1438 ± 62.6
PECA-2.5	1254 ± 11	17.4 ± 0.2
PECA-2.5-1	1185 ± 349	8.9 ± 0.9
PECA-5	1323 ± 137	3.34 ± 0.83
PECA-5-2	1097 ± 61	5.2 ± 1.65

Figure S3. The tensile stress strain curve of PECA (black), PECA-PPC blends with 2.5 and 5.0% of PPC, labeled PECA-2.5 and PECA-5, are represented in red and blue, respectively. While PECA-2.5 with 1.0% wt. of caffeic acid and PECA-5-2 with 2.0% wt. of caffeic acid, labeled

PECA-2.5-1 and PECA-5-2, are plotted in orange and in green, respectively. **(b)** Table with Young's modulus (MPa) and percent elongation values calculated for all blends.
